# Epidemiology of Community-acquired Bacteremia Among Children One to Fifty-nine Months of Age Admitted to a Tertiary Hospital in Harar, Eastern Ethiopia

**DOI:** 10.1097/INF.0000000000004842

**Published:** 2025-04-28

**Authors:** Yunus Edris, Desalegn A. Ayana, Alexander M. Aiken, Gezahang Mengesha, Faisel A. Hassen, Fami Ahmed, Dadi Marami, Belete Getnet, Nega Assefa, J. Anthony G. Scott, Lola Madrid

**Affiliations:** From the * Department of Infectious Disease Epidemiology and International Health, London School of Hygiene & Tropical Medicine, London, United Kingdom; † College of Health and Medical Sciences, Haramaya University, Harar, Ethiopia; ‡ KEMRI-Wellcome Trust Research Programme, Kilifi, Kenya.

**Keywords:** children, *Klebsiella oxytoca*, bacteremia, surveillance, child mortality, Ethiopia

## Abstract

**Background:**

Community-acquired bacteremia is a leading cause of mortality in children <5 years of age in Ethiopia, yet data on etiology are scarce. We described the etiology and risk factors for bacteremia and in-hospital mortality in a tertiary hospital in eastern Ethiopia.

**Methods:**

Clinical surveillance was conducted at Hiwot Fana Comprehensive Specialized Hospital from December 2021 to November 2023. All admitted children 29 days to 59 months old were eligible for blood culture collection, excluding elective surgery or poisoning.

**Results:**

Of 3384 admissions, 2366 were sampled; 2070 had uncontaminated blood cultures, and 236 (11.4%) had bacteremia. The incidence risk was 69.7 per 1000 admissions. *Klebsiella oxytoca* (n = 59, 25.0%) and *Klebsiella pneumoniae* (n = 30, 12.7%) were the most common pathogens. The leading Gram-positive pathogen was *Streptococcus pneumoniae* (n = 16, 6.8%). Gram-negative bacteria showed high resistance to ampicillin and gentamicin. Of 2070, 122 died, yielding a case fatality ratio of 13.1% in bacteremia cases compared to 5.0% in nonbacteremic cases. Severe wasting [adjusted odds ratio, 1.49, (95% confidence interval: 1.10–2.01)] was associated with bacteremia risk. Bacteremic cases had a high risk of death across all nutritional statuses, while nonbacteremic admissions exhibited increased mortality risk with the severity of the nutritional status.

**Conclusion:**

A high proportion of children admitted to Hiwot Fana Comprehensive Specialized Hospital had bacteremia with attendant high mortality. *K. oxytoca* was the commonest cause, showing significant resistance to first-line antimicrobials.

In 2022, the World Health Organization (WHO) estimated there were 4.9 million deaths globally among children <5 years of age. Most of these deaths happened in sub-Saharan Africa. In Ethiopia and other low and middle-income countries (LMICs), bacteremia, complications of prematurity and birth asphyxia are reported as the leading causes of mortality.^1,2^ The etiology of bacteremia varies based on time, geography,^3^ and age group.^4^ Gram-positive bacteria are typical causative agents of childhood bacteremia in high-income countries.^5^ In contrast, in LMICs, Gram-negative bacteria, especially *Klebsiella pneumoniae*, are the main etiologic causes^3,6^ whilst *Streptococcus pneumoniae* and *Staphylococcus aureus* are relatively rare. The overall case fatality ratio (CFR) of bacteremia in African children is around 15%, though this varies with host, pathogen and contextual factors.^4,7,8^

Estimates of the burden of bacteremia and the antibiotic susceptibility patterns of significant pathogens in children 1–59 months old in LMICs are scarce.^4^ This is partly due to limited access to suitable diagnostic microbiology facilities. For example, in Ethiopia, between 2013 and 2024, we identified only 45 studies that examined childhood bacteremia, with only 4 focusing on children beyond the neonatal period.^9–15^ Two of the 4 studies were conducted in Addis Ababa, the capital city, where 3 of the 6 accredited blood culture laboratories are located.^16^ Furthermore, these studies shared various limitations, including small sample sizes, exclusive focus on the intensive care unit admissions^14^ or inclusion of likely culture contaminants as pathogens (eg, single isolations of Coagulase-negative *staphylococci*).^15^ To optimize a treatment guideline and inform control strategies in Ethiopia and other similar settings, there is a need for a better understanding of the contemporary distribution and risk factors for principal bacteremia pathogens.^4,17^

We aimed to describe the etiology and mortality of community-acquired bacteremia among children 29 days to 59 months old admitted at a tertiary hospital in Harar, eastern Ethiopia.

## METHODS AND MATERIALS

### Study Setting

The study was conducted at Hiwot Fana Comprehensive Specialized Hospital (HFCSH), Harar, within the Harari Regional State in Ethiopia, 525 km east of the capital city of Addis Ababa. Harar City has a population of 276,000 people, and the HFCSH is a university teaching hospital serving a catchment area of ~5.8 million people. HFCSH and the regional laboratory in Harar are the only facilities in eastern Ethiopia currently offering bacterial culture. For children above the neonatal age, there are 89 beds with approximately 1700 admissions/year. Currently, common nonexclusive causes of beyond neonatal pediatric admission at HFCSH are the clinical diagnosis of pneumonia (~60%), malnutrition (~40%), meningitis (~10%) and tuberculosis (~5%). In Ethiopia, vaccinations against tuberculosis, polio, pentavalent, rotavirus, pneumococcus and measles are available, with an average full coverage of 39%, according to a survey conducted in eastern Ethiopia from 2017 to 2021.^18^

### Study Design and Participants

We conducted clinical surveillance that included all children 29 days to 59 months of age admitted to HFCSH between December 1, 2021 and November 30, 2023. Children were excluded if the reason for admission was elective surgery or poisoning, consent was denied, or a blood sample was not collected within 48 hours of admission.

We defined acute admissions as medical conditions requiring immediate inpatient care. Severe wasting was defined as weight for a length z-score of < −3 or bilateral pitting edema, while weight for a length of between −2 and −3 was categorized as moderate wasting.^19^ Clinically suspected bacteremia was considered if the clinician diagnosed focal sepsis (including but not limited to pneumonia, meningitis and soft tissue abscess) or if the child had a fever (≥38 °C) or hypothermia (<35.5 °C) at admission.^19,20^ Anemia was diagnosed if hemoglobin concentration measured on admission was <11 g/dL.^21^

In HFCSH, clinicians follow the WHO and Ethiopian treatment guidelines, which recommend ampicillin and gentamicin combination as first-line treatment of suspected bacteremia^19,22^, and subsequently modified by clinical response and laboratory results. However, for nonseverely wasted children >3 months old, we use ceftriaxone alone as a first line owing to better affordability. Ciprofloxacin and meropenem are reserved for severe sepsis. Meropenem is not usually available in the hospital and only can be given if the family can afford to purchase it privately. Data on age, sex, vaccination status (verbally reported by caregivers), clinical parameters at admission and outcome at discharge were collected. Enrolled children were followed up throughout their stay in the hospital until discharge or death.

### Blood Sample Collection and Laboratory Analysis

Blood samples were collected from eligible patients at admission or allowing a maximum of 2 days thereof. Samples were inoculated into BACT/ALERT PF Plus (bioMérieux, Inc., Durham, NC) culture bottles and processed with the automated BACT/ALERT incubator for 5 days. If growth was detected, a Gram stain was performed and subcultured on chromogenic, blood, chocolate, MacConkey and Xylose-Lysine-Deoxycholate agar and incubated at 37 °C in aerobic and CO2-enriched conditions. The suspected pathogens were identified at the species level using the analytical profile index biochemical identification kits (bioMérieux) as per the manufacturer’s instructions. *Micrococcus* spp., *Bacillus* spp., *Diphtheroids*, *Corynebacterium* spp., Coagulase-negative *staphylococci*, *Propionibacterium*,^8^ and unidentified Gram-positive rods were considered contaminants for all samples. We included the identification of *Candida* spp. from blood culture under the term “bacteremia” and considered these to be pathogens.

Antimicrobial susceptibility testing was performed using the Kirby-Bauer disc diffusion method with Oxoid diffusion discs (Thermo Fisher, Waltham, MA) according to the Clinical Laboratory Standard Institute guidelines.^23^ The intermediate-susceptibility status group was grouped with resistant isolates. The quality controls used were the appropriate American Type Culture Collection controls. The laboratory is ISO 15189 accredited and undergoes regular external quality control assessment by the UK National External Quality Assessment Service.

### Statistical Analysis

All analyses were performed using STATA SE version 18. We used descriptive statistics to summarize the child’s demography and clinical condition at admission, treatment outcomes and identified pathogens.

We then undertook 2 multivariable analyses: (i) risk factors for the presence of bacteremia and (ii) risk factors for death following bacteremia. For simplicity, we excluded polymicrobial bacteremia cases from these analytic models as we felt they represented a separate clinical entity. A binomial family regression model with logit link was used to assess the association between the exposure variables and bacteremia on admission compared with nonbacteremic admissions. Model outputs of the risk factor analysis are presented as odds ratios with 95% confidence intervals (CIs). The impact of bacteremia on inpatient mortality was evaluated using binomial family regression models with a log link, and model outputs were presented as risk ratios with 95% CIs.

In both modeling sets, variables with a *P* < 0.1 in the univariate analysis were included in the multivariable model. Sex and the dichotomized age groups-an infant (29 days to 11 months) and an older child (12–59 months) were retained in both models because these were reported in prior studies to have a biological influence on the 2 outcomes: bacteremia or in-hospital mortality. We looked for interactions between relevant variables in the resulting multivariable models.

This study was approved by the Research Ethics Review Committee at Haramaya University’s College of Health and Medical Sciences and the London School of Hygiene and Tropical Medicine (Reference 14394–3). For each child investigated, a parent/guardian provided written informed consent.

### Funding Source

This work was supported by the Bill & Melinda Gates Foundation (Grant reference numbers OPP1126780 and 100548). The funding organizations provided input on study design but had no role in data collection, analysis or interpretation.

## RESULTS

**FIGURE 1.**
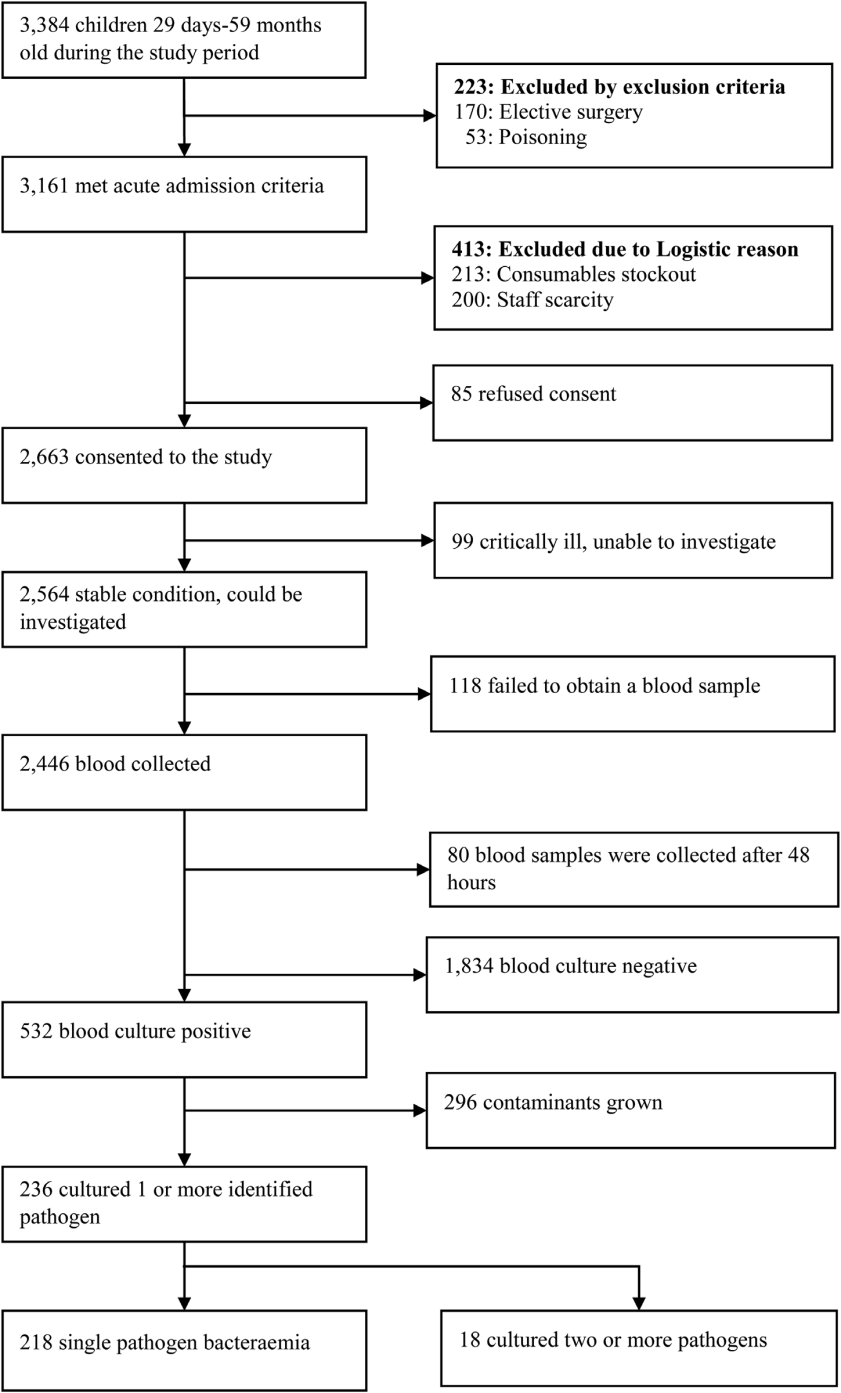
Flow diagram of children 29 days–59 months of age admitted to Hiwot Fana Comprehensive Specialized Hospital between December 1, 2021 and November 30, 2023.

From December 1, 2021 to November 30, 2023, 3384 children 29 days–59 months old were hospitalized in HFCSH; of these, 3161 (93.4%) children were acute admissions. Consent was denied for 85 (2.7%) of the children, and for 217 (6.9%) of the consented cases, the sample was not collected on admission due to critical illness and difficulty of venesection. Blood was collected for culture in 2,446 (72.3% of all admissions, Fig. 1), of whom 80 (2.5%) were collected after 48 hours and excluded from this analysis. The baseline characteristics and the pathogens identified in these excluded cases are detailed elsewhere (see Figure, Supplemental Digital Content 1, https://links.lww.com/INF/G210 and Table, Supplemental Digital Content 2, https://links.lww.com/INF/G211, respectively). Of 2366 blood cultures conducted, 296 (12.5%) grew organisms defined as contaminants and excluded from later analysis (Fig. 1). After excluding the 296 considered contaminants, 11.4% (236/2070) grew at least one pathogen, including 18 samples where multiple pathogens were cultured simultaneously. The analysis dataset thus comprised 2070 children: 1834 with negative blood cultures and 236 with one or more identified pathogens on blood culture.

Among this set of 2070 children, 867 (41.9%) were female, 488 (23.6%) were not vaccinated at all, 929 (44.9%) had acute malnutrition, 724 (35.0%) were severely wasted and 205 (9.9%) had moderate wasting. Additionally, 533 (25.7%) had anemia and 1458 (70.4%) were admitted with the clinically suspected bacteremia diagnosis (Table 1).

**TABLE 1.** Baseline Characteristics of Children 29 Days–59 Months Old Stratified by the Blood Culture Results (n = 2070)

Characteristics	Bloodstream Infection, n (%)			
	Nonbacteremic	Monopathogens	Polymicrobial	Total
Age				
29 days–11 months	749 (40.8%)	100 (45.9%)	8 (44.4%)	857 (41.4%)
12–59 months	1085 (59.2%)	118 (54.1%)	10 (55.6%)	1213 (58.6%)
Sex				
Female	771 (42.0%)	87 (39.9%)	9 (50.0%)	867 (41.9%)
Male	1063 (58.0%)	131 (60.1%)	9 (50.0%)	1203 (58.1%)
Vaccination status				
Completely vaccinated	994 (54.2%)	105 (48.2%)	7 (38.9%)	1106 (53.4%)
Partially vaccinated	336 (18.3%)	40 (18.3%)	6 (33.3%)	382 (18.5%)
Not vaccinated	422 (23.0%)	61 (28.0%)	5 (27.8%)	488 (23.6%)
Unknown	82 (4.5%)	12 (5.5%)	0 (0.0%)	94 (4.5%)
Season				
October–January (dry)	449 (24.5%)	53 (24.3%)	5 (27.8%)	507 (24.5%)
February–May (mild rainy)	548 (29.9%)	76 (34.9%)	5 (27.8%)	629 (30.4%)
June–September (heavy rainy)	837 (45.6%)	89 (40.8%)	8 (44.4%)	934 (45.1%)
Clinically suspected bacteremia at admission				
Not suspected	458 (25.0%)	55 (25.2%)	3 (16.7%)	516 (24.9%)
Suspected	1289 (70.3%)	155 (71.1%)	14 (77.8%)	1458 (70.4%)
Unknown	87 (4.7%)	8 (3.7%)	1 (5.6%)	96 (4.6%)
Nutritional status				
Well-nourished	1027 (56.0%)	103 (47.2%)	11 (61.1%)	1141 (55.1%)
Moderate wasting	179 (9.8%)	24 (11.0%)	2 (11.1%)	205 (9.9%)
Severe wasting	628 (34.2%)	91 (41.7%)	5 (27.8%)	724 (35.0%)
Hemoglobin level				
Nonanemic	1356 (73.9%)	167 (76.6%)	14 (77.8%)	1537 (74.3%)
Anemic (<11 mg/dL)	478 (26.1%)	51 (23.4%)	4 (22.2%)	533 (25.7%)
Outcome				
Survived	1743 (95.0%)	191 (87.6%)	14 (77.8%)	1948 (94.1%)
Died	91 (5.0%)	27 (12.4%)	4 (22.2%)	122 (5.9%)

### Type of Bacteremia on Admission and Associated Risk Factors

Based on the total number of admissions in the study period (n = 3384, including those not recruited into surveillance), a minimum incidence risk of 69.7 bacteremia episodes per 1000 admissions (236/3384) was estimated. Among 236 children with pathogenic bacteremia (both monomicrobial and polymicrobial), *Klebsiella oxytoca* (n = 59, 25.0%) and *K. pneumoniae* (n = 30, 12.7%) were the most common pathogens. Gram-positive pathogens were few, the commonest being *S. pneumoniae* (n = 16, 6.8%) and *S. aureus* (n = 12, 5.1%). In contrast to our work on neonatal bacteremia in the same hospital, *Pantoea agglomerans* were relatively rare in this age group (n = 6, 2.5%) (manuscript under review). Other isolates are detailed in Table 2.

**TABLE 2.** Pathogens Isolated in 236 Children 29 Days–59 Months of Age Enrolled in the Study

Pathogens Identified	Episodes of Bacteremia by Age Group and Overall Incidence				Overall Outcome		
	Total (n)	29 Days–11 Months	12–59 Months	Bacteremia Incidence/1000 Admissions	Death (n)	CFR (%)	CFR 95% CI
Single pathogen infections	218	100	118	64.4	27	12.4	8–17
Gram-negatives	154	69	85	45.5	22	14.3	9–20
*Klebsiella oxytoca*	51	20	31	15.1	6	12	NA
*Klebsiella pneumoniae*	26	16	10	7.7	1	4	NA
*Serratia* species	18	9	9	5.3	3	17	NA
*Escherichia coli*	15	9	6	4.4	7	47	19–68
*Burkholderia cepacia*	12	6	6	3.5	2	17	NA
*Pantoea agglomerans*	6	3	3	1.8	0	0.0	NA§
Other Gram-negatives*	26	6	20	7.7	3	12	NA
Gram-positives	55	27	28	16.3	4	7.3	0.0–13
*Staphylococcus aureus*	11	4	7	3.3	1	9	NA
*Streptococcus pneumoniae*	16	6	10	4.7	3	19	NA
*Enterococcus* species	11	7	4	3.3	0	0.0	NA
Viridians group *Streptococci*	9	6	3	2.7	0	0.0	NA
Other Gram-positives†	8	4	4	2.4	0	0.0	NA
Fungal infections	9	4	5	2.7	1	11.1	NA
Candida species	9	4	5	2.7	1	11.1	NA
Polymicrobial infections‡	18	8	10	5.3	4	22.2	3–41
Any bacteremia	236	108	128	69.7	31	13.0	9–17

* This group includes 6 in 29 days–11 months which are *Moraxella catarrhalis* (1), *Pseudomonas mendacina* (1), *Salmonella* spp. (2), *Sphingobacterium spiritivorum* (1), *Enterobacter cloacae* (2) and 20 in 12 months–59 months are *Acinetobacter iwoffii* (1), *Stenotrophomonas maltophilia* (1), *Acinetobacter baumannii* (2), *Enterobacter cloacae* (2), *Kocuria varians* (1), *Kocuria kristinae* (2), *Neisseria meningitidis* (1), *Pasteurella pneumoniae* (1), *Proteus mirabilis* (1), *Salmonella enterica* (7), *Haemophilus influenzae* (1).

† This group includes 4 in 29 days–11 months age group which are: Group B *Streptococcus* (1), Group C *Streptococcus* (2) and Group G *Streptococcus* (1) while also 4 in 12 months–59 months are Viridans group streptococci (1), Group G *Streptococcus* (1) and *Streptococcus mitis* (2).

‡ In 29 days to 11 months age group are *Klebsiella pneumoniae* + *Escherichia coli* (1), *Klebsiella pneumoniae* + *Pantoea agglomerans* (1), *Klebsiella oxytoca* + *Acinetobacter baumannii* (1), *Serratia* spp. + *Klebsiella oxytoca* (1), *Escherichia coli* + *Salmonella* spp. (1), Group G *Streptococcus* + *Burkholderia cepacia* (1) and *Burkholderia cepacia* + *Klebsiella oxytoca* (1). In 12 to 59 months age group are *Klebsiella oxytoca* + *Acinetobacter baumannii* (1), *Klebsiella oxytoca* + *Serratia* spp. (3), *Enterobacter gergoviae* + *Enterobacter cloacae* (1), *Enterobacter cloacae* + *Klebsiella oxytoca* (1), *Enterococcus faecalis* + *Streptococcus bovis* (2) and *Salmonella* spp. + *Pantoea agglomerans* (1).

§ We did not calculate the 95% CI for the CFR of the isolates in which the number of hospital deaths is not at least 4 or above as indicated by the NA, for not applicable in the table.

CI indicates confidence interval; CRF, case fatality ratio.

We examined risk factors for monomicrobial infection (n = 218) by comparison with nonbacteremia patients (n = 1834). In multivariable analyses, only severe wasting [adjusted odds ratio: 1.49, (95% CI: 1.10–2.01)], was associated with the presence of bacteremia (Table 3). The point estimate for moderate wasting was also elevated, but confidence intervals were wide [adjusted odds ratio: 1.38, (95% CI: 0.86–2.23)].

**TABLE 3.** Univariate and Multivariate Analysis of Factors Associated With Bacteremia Compared to Nonbacteremic Children 29 Days–59 Months Old (N = 2036)

Exposure Variable	Univariate Analysis			Multivariate Analysis		
	OR*	(95% CI)	*P* Value	aOR†	(95% CI)	*P* Value
Age group						
29 days–11 months	1.23	0.93–1.63	0.15	1.28	0.96–1.71	0.09
Sex						
Male	1.09	0.82–1.45	0.55	1.09	0.82–1.46	0.54
Season of admission						
February–May (mild rainy)	1.17	0.81–1.70	0.81			
June–September (heavy rainy)	0.90	0.63–1.29	0.57			
Nutritional status						
Moderate wasting	1.34	0.83–2.14	0.23	1.38	0.86–2.23	0.18
Severe wasting	1.44	1.07–1.95	**0.02**	1.49	1.10–2.01	**0.01**
Hemoglobin level						
Anemia	0.87	0.62–1.21	0.39			

The reference groups were: age- 12 months–59 months, sex-female, season- October–January (dry season), Nutritional status- well-nourished and Hemoglobin level- nonanemic. Boldface was used to highlight the variable with a significant *P*-value.

* Odds ratio: Both the Unadjusted and Adjusted models were analyzed using a binomial family with a logit link regression model.

† aOR, adjusted odds ratio. Adjusted for the age group, Sex, season of the year, nutritional status and Hemoglobin level.

CI indicates confidence interval; OR, crude odds ratio.

### Inpatient Mortality and Associated Risk Factors

Among the 2070 children studied, 122 (5.9%) died in the hospital. The CFR varied depending on the presence of bacteremia (*P* < 0.0001, Table, Supplemental Digital Content 3, https://links.lww.com/INF/G212). For nonbacteremic cases, the CFR was lower at 5.0% (91/1834) compared to bacteremic cases, which had a CFR of 13.1% (31/236). The CFR for each organism is listed in Table 2.

In the univariable model of risk factors for mortality in monomicrobial bacteremia, 2 factors, wasting and bacteremia, were significant predictors of in-hospital death (Table, Supplemental Digital Content 4, https://links.lww.com/INF/G213).

The association between the severity of nutritional status and risk of death differs by bacteremia status (likelihood ratio test; *P* < 0.006). Among nonbacteremic children, the risk of in-hospital death was increased for those with moderate wasting [stratum-specific risk ratio (ssRR): 2.48, 95% CI: 1.20–5.14] and severe wasting (ssRR: 4.12, 95% CI: 2.56–6.62), compared to well-nourished children. Conversely, the variation in the risk of death by nutritional status was minimal among the bacteremia cases. However, they had a higher risk of death across all nutritional statuses compared to nonbacteremia (Table 4).

**TABLE 4.** Summary of Stratum-specific Risk Ratios for the Interaction of Nutritional Status and Bacteremia on In-hospital Mortality Among Acutely Admitted Children 29 Days–59 Months Old (N = 2052)

	Effect of Bacteremia on In-hospital Mortality, Stratified by the Nutritional Status								
	Well-nourished			Moderate Wasting			Severe Wasting		
Blood Culture Results	ssRR	(95% CI)	*P* value	ssRR	(95% CI)	*P* value	ssRR	(95% CI)	*P* value
Nonbacteremia	Reference			2.48	1.20–5.14	0.01	4.12	2.56–6.62	<0.0001
Bacteremia	5.63	2.94–10.78	<0.0001	5.57	1.79–17.30	<0.0001	5.41	2.72–10.73	<0.0001

The model was adjusted for the age group, sex and Hemoglobin level and it was analyzed using a binomial family with a log link regression model.

The reference groups were: age- 12 months–59 months, sex-female, Nutritional status- well-nourished, Hemoglobin level- nonanemic and blood culture results – nonbacteremic.

CI indicates confidence interval; ssRR, stratum-specific risk ratio.

### Antibiotic Susceptibility Patterns of Major Pathogens

We described the antibiotic susceptibility profiles of the 4 most common Gram-negative bacterial isolates, namely *K. oxytoca*, *K. pneumoniae*, *Serratia* spp. and *E. coli*. There were at least 10 isolates of each of these species. All these tested Gram-negative were resistant to ampicillin (100% of isolates tested). Gentamicin resistance varied, with 63.0% (32/51) of *K. oxytoca*, 38.0% of *K. pneumoniae* (8/21) and 70.0% (7/10) of *E. coli* being resistant. Similarly, 93.0% (40/43) of *K. oxytoca*, and 89.0% (8/9) of *K. pneumoniae* were resistant to ceftriaxone. Resistance to ciprofloxacin was 42.0% (21/50) in *K. oxytoca* and 48.0% (10/21) in *K. pneumoniae*. Both *K. oxytoca* and *K. pneumoniae* demonstrated frequent susceptibility to amikacin and meropenem (Table, Supplemental Digital Content 5, https://links.lww.com/INF/G214).

## DISCUSSION

This study of community-acquired bacteremia in non-neonatal children provides a comprehensive picture of the etiology at a tertiary hospital with a large catchment area in eastern Ethiopia, where previous clinical and microbiologic data are sparse. We observed a high burden of bacteremia and high associated mortality among hospitalized children. The frequent identification of *K. pneumoniae*-related bacteremia aligns with publications from other LMICs.^6^ Typically, *S. pneumoniae* has also been reported to be a major pathogen in African children,^3^ but this organism was relatively infrequently isolated in this setting with moderate local pneumococcal vaccination levels.^18^ However, in our hospital, *K. oxytoca* was unexpectedly the commonest cause of bacteremia in this age group, with a substantial contribution from a wide range of other Gram-negative organisms. Severe wasting was associated with bacteremia on admission. The risk of in-hospital death was significantly higher across all nutritional statuses among bacteremic children compared to nonbacteremic acute admissions. Interestingly, an interaction term suggested that the risk of death among severely wasted nonbacteremic children compared to nonbacteremic well-nourished children was pronounced. In contrast, this gradient of death by nutritional status was slight among bacteremic cases. We discuss possible explanations for this interaction below.

The observed 11.4% bacteremia proportion among tested samples is lower than the 15.5% (8.4%–24.4%) pooled estimate in Africa.^3^ This difference likely arises from the inclusion criteria of the 17 reviewed studies, where only 2 enrolled all admissions, reporting 5.9% in Kenya (1998–2002)^24^ and 8.0% in Mozambique (2001–2006).^25^ Additionally, other surveillance studies focused solely on children with suspected bacteremia,^26^ a factor that affects bacteremia proportion.^7^ This scenario indicates the scarcity of inclusive surveillance data in LMICs and underscores the representativeness of our findings for all acute admissions, minimizing selection bias.

*K. oxytoca* has rarely been reported in LMICs,^27^ while *K. pneumoniae* is a well-recognized pathogen among African children.^6^ Most literature documenting *K. oxytoca’*s pathogenicity and outbreak potential are case reports^28^ and limited studies from high-income countries.^29^ To check our laboratory methods, we used Microscan (Microscan Systems, Inc., 2021) to reidentify 16 randomly selected isolates of *K. oxytoca*, yielding 100% concordance. The high number of *K. oxytoca* bacteremia in the HFCSH indicates a potential shift in the landscape of bacterial profiles in this population. Comparison with a recent study in HFCSH conducted between March and November 2021^30^ showed only 2 cases of *K. oxytoca* in this age group over 8 months.^30^ In contrast, this current study identified 59 cases of *K. oxytoca* over 2 years [December 2021 to November 2023 (Table, Supplemental Digital Content 6, https://links.lww.com/INF/G215). This suggests an approximately 10-fold increase in *K. oxytoca* bacteremia frequency in a few years]. Both *Klebsiella* species, especially *K. oxytoca*, exhibited substantial resistance to first- and second-line antimicrobials relevant to our setting reflecting a concerning trend in antimicrobial resistance, as seen elsewhere.^31^

We found that community-acquired bacteremia was linked to severe wasting, aligning with previous studies.^24,32^ These findings underscore the importance of considering bacteremia in this group as prompt clinical diagnosis is often difficult due to unremarkable symptoms and signs of infections in malnourished children.^33^

Among nonbacteremic patients, nutritional status had a gradient effect on mortality; the chance of in-hospital death was higher in severely wasted (9.2%, 95% CI: 7.0–11.5) or moderately wasted (5.6%, 95% CI: 2.2–9.0) compared to well-nourished children (2.2%, 95% CI: 1.3–3.1). However, our findings suggest that once a child develops bacteremia, the risk of death is little influenced by the child’s nutritional status, varying from 12.1% to 12.6% (Tables, Supplemental Digital Content 7, https://links.lww.com/INF/G216 and 8, https://links.lww.com/INF/G217). Possible explanations for this finding include: First, severely wasted children may have a higher risk of death of nonidentified bacteremia, as they are more likely to receive antibiotic treatment prior to hospitalization, decreasing the already poor blood culture sensitivity. Secondly, severely wasted children with medical complications, suspected bacteremia in this case, are hospitalized more frequently for more intensive treatment in line with a national guideline.^22^ This may be reinforced by the payment-free treatment for severely wasted children <5 years of age in Ethiopia that alleviates economic barriers to health care access, specifically for this group.^34^ This scenario may introduce Berkson’s bias, as the study population may not accurately represent the broader population of children in the catchment area. Thirdly, the very large inflammatory response induced by bacteremia might dominate any smaller effects of immunodeficiency that might occur between well and poorly nourished children. Finally, survivor bias is also a possibility; severely wasted children who survive to reach the hospital may represent the more resilient group.

The main clinical implication of this finding underscores the crucial role of bacteremia in the in-hospital mortality risk of acutely admitted children, emphasizing the need for assessments for bacteremia, particularly among those presenting with moderate or severe wasting.

This study has some limitations. First, it is a single center and facility-based, which might limit its generalizability, although it is amongst the largest studies of its kind in Ethiopia. From a laboratory point of view, although this is a laboratory working to international quality criteria, the absence of anaerobic incubation conditions and the possible prior antibiotic use due to being a tertiary hospital receiving referral cases might have led to missed cases of bacteremia, possibly leading to an underestimation of the true bacteremia burden. Additionally, the blood culture contamination rate of 12.5% is far from the suggested benchmark of <3%,^35^ though it is similar to values reported from other LMICs^36,37^ – this might also have led to underestimation. Finally, the number of isolates available for antimicrobial susceptibility testing was relatively modest for some organisms, limiting the conclusions drawn regarding antimicrobial resistance patterns.

## CONCLUSION

Among children, 1–59 months of age admitted to a tertiary hospital in eastern Ethiopia, a substantially higher proportion were bacteremic compared to other published hospital-based studies in Africa. *K. oxytoca* was the single most common pathogen causing community-acquired bacteremia in this setting, whilst vaccine-preventable bacteria including *S. pneumoniae* and *H. influenzae type b* were rare. *K. pneumoniae* and *K. oxytoca* isolates at this hospital typically exhibited resistance to WHO-recommended first- and second-line antibiotic regimens. Wasting was both common and a significant risk factor for bacteremia. Unexpectedly, the anticipated gradient effect of nutritional status on in-hospital mortality was small amongst bacteremia patients compared to nonbacteremic admissions.

## ACKNOWLEDGMENTS

*We thank all the participants and their guardians, the clinicians attending the patients, and the HFCSH administrations, who have supported and participated in the research. JAGS is funded by The Wellcome Trust (214320).*

## Supplementary Material


