# Supplementary material for: Epidemiology of Community-acquired Bacteremia Among Children One to Fifty-nine Months of Age Admitted to a Tertiary Hospital in Harar, Eastern Ethiopia
Source: Pediatr Infect Dis J. 2025 Apr 28;44(10):913–9. doi: 10.1097/INF.0000000000004842 (PMC12422626; doi:10.1097/INF.0000000000004842)
Supplement: Supplementary file 1 [file inf-44-0913-s001.pdf]

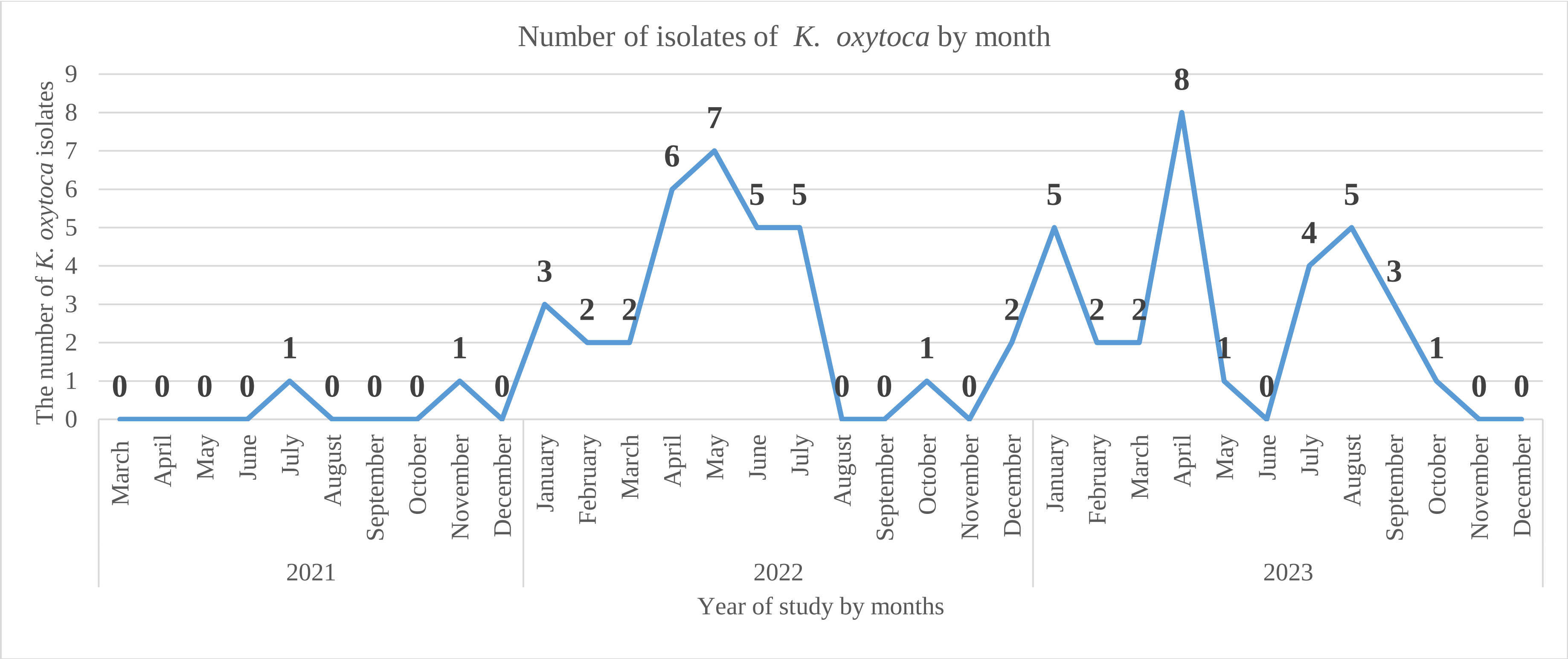

Supplemental Digital Content 1: Line graph of the Number of isolates of *K. oxytoca* by month from March 2021 to November 2023 in the HFCSH as identified by the MBIRA and current surveillance.

MBIRA: Mortality from Bacterial Infections Resistant to Antibiotics- It is a multinational prospective cohort study on the impact of antimicrobial resistance on mortality and cited 31st in the manuscript.
