# Supplementary material for: Epidemiology of Community-acquired Bacteremia Among Children One to Fifty-nine Months of Age Admitted to a Tertiary Hospital in Harar, Eastern Ethiopia
Source: Pediatr Infect Dis J. 2025 Apr 28;44(10):913–9. doi: 10.1097/INF.0000000000004842 (PMC12422626; doi:10.1097/INF.0000000000004842)
Supplement: Supplementary file 2 [file inf-44-0913-s002.pdf]

Supplemental Digital Content 2: Baseline characteristics of acute admissions aged 29 days-59 months for which blood culture was collected beyond 48 hours of admission stratified by the blood culture result (n=71*) and participants who consented, but we could not obtain their blood (n=217).

Characteristics	Bloodstream infection (N=71), n (%)			Consented, but not blood sampled (N=217), n (%)		
	Non-bacteremic	Monopathogens	Total	Critical	Vein not found	Total
**Age**						
29 days-11 months	27 (46.6%)	9 (69.2%)	36 (50.7%)	43 (43.4%)	59 (50.0%)	102 (47.0%)
12-59 months	31 (53.4%)	4 (30.8%)	35 (49.3%)	56 (56.6%)	59 (50.0%)	115 (53.0%)
**Sex**						
Female	23 (39.7%)	2 (15.4%)	25 (35.2%)	37 (37.4%)	47 (39.8%)	84 (38.7%)
Male	35 (60.3%)	11 (84.6%)	46 (64.8%)	62 (62.6%)	71 (60.2%)	133 (61.3%)
**Vaccination status**						
Completely vaccinated	27 (46.6%)	6 (46.2%)	33 (46.5%)	48 (48.5%)	59 (50.4%)	107 (49.5%)
Partially vaccinated	16 (27.6%)	0 (0.0%)	16 (22.5%)	20 (20.2%)	27 (23.1%)	47 (21.8%)

Not vaccinated	13 (22.4%)	6 (46.2%)	19 (26.8%)	26 (26.3%)	29 (24.8%)	55 (25.5%)
Unknown	2 (3.4%)	1 (7.7%)	3 (4.2%)	5 (5.1%)	2 (1.7%)	7 (3.2%)
**Season**						
Oct-Jan (dry)	19 (32.8%)	4 (30.8%)	23 (32.4%)	27 (27.3%)	34 (28.8%)	61 (28.1%)
Feb-May (mild rainy)	16 (27.6%)	7 (53.8%)	23 (32.4%)	36 (36.4%)	30 (25.4%)	66 (30.4%)
Jun-Sep (heavy rainy)	23 (39.7%)	2 (15.4%)	25 (35.2%)	36 (36.4%)	54 (45.8%)	90 (41.5%)
**Clinically suspected bacteremia diagnosis at admission**						
Not suspected	4 (20.0%)	0 (0.0%)	4 (16.0%)	2 (66.7%)	0 (0.0%)	2 (50.0%)
Suspected	16 (80.0%)	5 (100.0%)	21 (84.0%)	1 (33.3%)	1 (100.0%)	2 (50.0%)
**Nutritional status**						
Well-nourished	27 (46.6%)	6 (46.2%)	33 (46.5%)	43 (43.4%)	65 (55.1%)	108 (49.8%)
Moderate wasting	2 (3.4%)	2 (15.4%)	4 (5.6%)	10 (10.1%)	7 (5.9%)	17 (7.8%)
Severe wasting	29 (50.0%)	5 (38.5%)	34 (47.9%)	46 (46.5%)	46 (39.0%)	92 (42.4%)
**Hemoglobin level**						
Nonanemic	41 (70.7%)	6 (46.2%)	47 (66.2%)	70 (70.7%)	89 (75.4%)	159 (73.3%)
Anemic (<11mg/dl)	17 (29.3%)	7 (53.8%)	24 (33.8%)	29 (29.3%)	29 (24.6%)	58 (26.7%)

**Outcome**						
Survived	54 (93.1%)	11 (84.6%)	65 (91.5%)	76 (76.8%)	103 (87.3%)	179 (82.5%)
Death	4 (6.9%)	2 (15.4%)	6 (8.5%)	23 (23.2%)	15 (12.7%)	38 (17.5%)

*We have indicated in the manuscript that we excluded 80, but only 71 are described here as nine were considered contaminants as per defined in the manuscript. We focused on the potential pathogens and no growths, the same approach as for those enrolled in the study. There were no polymicrobial results. Notably, there were missing values for both those sampled after 48 hours and those 217 consented not sampled group. One missing value for the age group and 46 for clinically suspected bacteremia diagnosis in the 71 group, while there was one missing value for the vaccination status and 213 in the 217 group for the clinically suspected bacteremia diagnosis at admission.
