# Supplementary material for: Epidemiology of Community-acquired Bacteremia Among Children One to Fifty-nine Months of Age Admitted to a Tertiary Hospital in Harar, Eastern Ethiopia
Source: Pediatr Infect Dis J. 2025 Apr 28;44(10):913–9. doi: 10.1097/INF.0000000000004842 (PMC12422626; doi:10.1097/INF.0000000000004842)
Supplement: Supplementary file 3 [file inf-44-0913-s003.pdf]

Supplemental Digital Content 3: Pathogens isolated in 13 children aged 29 days-59 months enrolled in the study for which blood culture was collected after 48 hours of admission.

Pathogens identified	Total (n)	29 days-11 months	12-59 months	Death (n)
**Single pathogen infections**	**13**	**9**	**4**	**2**
**Gram-negatives**	**10**	**6**	**4**	**2**
Klebsiella oxytoca	5	3	2	0
***Serratia* species**	2	2	0	0
Escherichia coli	1	1	0	0
Kocuria kristinae	2	0	2	2
**Gram positive**	3	3	0	0
Staphylococcus aureus	1	1	0	0
***Enterococcus* species**	2	2	0	0

*Among the 71 cases for whom we collected blood samples after 48 hours of admission, we identified clinically significant pathogens in only 13 blood cultures.
