# Supplementary material for: Epidemiology of Community-acquired Bacteremia Among Children One to Fifty-nine Months of Age Admitted to a Tertiary Hospital in Harar, Eastern Ethiopia
Source: Pediatr Infect Dis J. 2025 Apr 28;44(10):913–9. doi: 10.1097/INF.0000000000004842 (PMC12422626; doi:10.1097/INF.0000000000004842)
Supplement: Supplementary file 4 [file inf-44-0913-s004.pdf]

Supplemental Digital Content 4: The effect of blood culture results on in-hospital mortality among acutely admitted children aged 29 days-59 months (N=2,070).

**Blood culture result**	**Treatment outcome, n**			
	**Survived**	**Death, n (%)**	**Total**	**P value**
**No bacteremia**	1743	91 (5.0)	1834	
**Bacteremia**	205	31 (13.1)	236	**<0.0001**
**Total**	1948	122 (5.9)	2070	
