# Supplementary material for: Epidemiology of Community-acquired Bacteremia Among Children One to Fifty-nine Months of Age Admitted to a Tertiary Hospital in Harar, Eastern Ethiopia
Source: Pediatr Infect Dis J. 2025 Apr 28;44(10):913–9. doi: 10.1097/INF.0000000000004842 (PMC12422626; doi:10.1097/INF.0000000000004842)
Supplement: Supplementary file 5 [file inf-44-0913-s005.pdf]

Supplemental Digital Content 5: Univariate of factors associated with in-hospital mortality among acutely admitted children aged 29 days-59 months (N=2,052).

| | Univariate analysis | | |
|---|---|---|---|
| Exposure variable | RR | (95% CI) | P value |
| **Age group** | | | |
| 29 days-11 months | 0.91 | 0.63-1.30 | 0.59 |
| **Sex** | | | |
| Male | 0.82 | 0.58-1.17 | 0.28 |
| **Nutritional status** | | | |
| Moderate wasting | 2.01 | 1.09-3.72 | **0.03** |
| Severe wasting | 3.01 | 2.04-4.46 | **<0.0001** |
| **Hemoglobin level** | | | |
| Anemia | 1.12 | 0.76-1.65 | 0.58 |
| **Blood culture results** | | | |
| Bacteremic | 2.50 | 1.66-3.75 | **<0.0001** |

CI= confidence interval. *RR= crude risk ratio

The reference groups were: age- 12months-59months, sex-female, Nutritional status- well nourished, Hemoglobin level- non-anemic, and blood culture results – non-bacteremic.

†Risk ratio: Model was analyzed using a binomial family with a log link regression model
