# Supplementary material for: Epidemiology of Community-acquired Bacteremia Among Children One to Fifty-nine Months of Age Admitted to a Tertiary Hospital in Harar, Eastern Ethiopia
Source: Pediatr Infect Dis J. 2025 Apr 28;44(10):913–9. doi: 10.1097/INF.0000000000004842 (PMC12422626; doi:10.1097/INF.0000000000004842)
Supplement: Supplementary file 6 [file inf-44-0913-s006.pdf]

Supplemental Digital Content 6: Antimicrobial susceptibility profile of the four major isolates against commonly used antibiotics.

	Blood culture results			
**Antibiotics tested**	*K. oxytoca*	*K. pneumoniae*	*Serratia* spp	*E. coli*
Ampicillin				
Susceptible	0 (0.0%)		0 (0.0%)	0 (0.0%)
Resistant	50 (100.0%)	Not tested	16 (100.0%)	11 (100.0%)
Gentamicin				
Susceptible	19 (37.3%)	13 (61.9%)	12 (80.0%)	3 (30.0%)
Resistant	32 (62.7%)	8 (38.1%)	3 (20.0%)	7 (70.0%)
Ceftriaxone				
Susceptible	3 (7.0%)	1 (11.1%)	1 (6.2%)	0 (0.0%)

Resistant	40 (93.0%)	8 (88.9%)	15 (93.8%)	8 (100.0%)
Ceftazidime				
Susceptible	4 (7.8%)	5 (31.2%)	0 (0.0%)	1 (10.0%)
Resistant	47 (92.2%)	11 (68.8%)	15 (100.0%)	9 (90.0%)
Amoxicillin-clavulinic acid				
Susceptible	1 (2.0%)	9 (47.4%)	1 (6.7%)	2 (16.7%)
Resistant	50 (98.0%)	10 (52.6%)	14 (93.3%)	10 (83.3%)
Ciprofloxacin				
Susceptible	29 (58.0%)	11 (52.4%)	15 (93.8%)	8 (66.7%)
Resistant	21 (42.0%)	10 (47.6%)	1 (6.2%)	4 (33.3%)
Amikacin				
Susceptible	42 (93.3%)	20 (95.2%)	14 (93.3%)	9 (81.8%)
Resistant	3 (6.7%)	1 (4.8%)	1 (6.7%)	2 (18.2%)

Meropenem				
Susceptible	51 (100.0%)	20 (95.2%)	16 (100.0%)	9 (81.8%)
Resistant	0 (0.0%)	1 (4.8%)	0 (0.0%)	2 (18.2%)

The number of respective tested antibiotics does not match the total isolates for *K. oxytoca* (n=51), K. pneumoniae (n=26), Serratia spp. (n=18), and E. coli (n=26), as there was a missing number for the tested antibiotics. This was due to interruptions in the supply of susceptibility testing discs during the project
